# Chemical Composition and Crystallization Behavior of Oil and Fat Blends for Spreadable Fat Applications

**DOI:** 10.3390/foods13203305

**Published:** 2024-10-18

**Authors:** Maureen Gerlei, Hugo Pierson, Marc Ponçot, Cyril J. F. Kahn, Michel Linder

**Affiliations:** 1 Laboratoire d’Ingénierie des Biomolécules (LIBio), Université de Lorraine, F-54500 Vandœuvre-lès-Nancy, France; maureen.gerlei@univ-lorraine.fr (M.G.); cyril.kahn@univ-lorraine.fr (C.J.F.K.); 2 Institut Jean Lamour CNRS, Université de Lorraine, F-54000 Nancy, France; hugo.pierson@univ-lorraine.fr (H.P.); marc.poncot@univ-lorraine.fr (M.P.)

**Keywords:** anhydrous milk fat, lipid mixture, monoglycerides, diglycerides, thermal behavior, polymorphism transition, stop-and-return DSC, X-ray diffraction, Raman spectroscopy

## Abstract

To meet the expectations of European consumers, who prioritize agro-environmental factors and local resources, the substitution of fats (palm, coconut, shea) and achieving a balanced fatty acid profile in spreadable fats are gaining more attention. The crystallization at 4 °C of a lipid blend composed of rapeseed oil, anhydrous dairy fats, and emulsifiers was studied using a multi-scale approach (DSC and X-ray diffraction techniques) to understand the emergence of polymorphic structures. Although the addition of PUFA from rapeseed oil reduces the atherogenicity and thrombogenicity indices in the blend, controlling the cooling kinetics influences the shapes (needles and spherulites) and sizes of the crystalline structures (small crystals form at a cooling rate of 1 °C min^−1^, while larger crystals form at higher rates of 5 and 10 °C min^−1^). The crystallization behavior revealed differences in polymorphic forms at 4 °C in the blend, with a transition to different forms occurring more rapidly compared to dairy fat (stop-and-return method). The study shows crystalline coexistence (α, β′, and β) in a 2L lamellar structure, with the β′ form being predominant. This structure is ideal for formulating a spreadable product, offering good spreadability (SFC < 32% at 10 °C), mouthfeel, and nutritional benefits compared to butter.

## 1. Introduction

In 2023, France remains the world’s largest consumer of butter, with an annual consumption of 8.2 kg per capita, closely followed by Germany, according to the Centre national interprofessionnel de l’économie laitière (CNIEL (French Dairy Interbranch Organization)). Butter is mainly used in cooking, as an ingredient in pastries and baked goods, and is a breakfast staple, often spread on toast. Germany is also a major consumer of margarine, along with the Netherlands. Margarine consumption in France remains significantly lower than butter, with only 3.3 kg per household in 2019 as per l’Établissement national des produits de l’agriculture et de la mer (FranceAgriMer (The French National Establishment for Agricultural and Marine Products)). Over 2 million tons a year of “margarine and shortening” have been produced in Europe in recent years (FAOSTAT (The statistical database of the Food and Agriculture Organization (FAO) of the United Nations)). The emergence in recent years of fat spreads and blended spreads, containing not less than 10% and not more than 90% fat [1], offers certain advantages in terms of lipid formulation, texture and spreadability, unlike dairy butters, whose legislation remains highly regulated [2,3]. Indeed, they can only be produced using dairy cream and mechanical processes [4]. Margarine and spreadable fat are obtained from a mixture of oils, fats, with the addition of colorants, vitamins, salts, and emulsifiers. The emulsifiers stabilize the emulsion, which is made up of a fatty phase and a more or less substantial aqueous phase. Mono- and diacylglycerols are lipidic emulsifiers (E471), considered “GRAS” (generally recognized as safe) commonly used as enhancers of crystallization and nucleation properties [5,6]. The lipid formulations used enable the desired health claims to be met, particularly in terms of essential fatty acids, such as n-3 polyunsaturated fatty acids [4,7]. The use of anhydrous milk fat and its olein and stearin fractions to achieve desired dropping points is widely described in the literature according to their uses (spreadable fat, shortening, puff pastry, bakery product). Textural, functional and sensory properties are highly dependent on the structure of the crystal as a function of the temperature studied [8,9,10].

The goal is to replace fats not produced in Europe (such as palm oil, shea butter, or coconut oil) with dairy fats (AMF and stearin) to reduce the carbon footprint and meet consumer expectations, as they increasingly consider the agro-environmental aspect and seek to use local resources.

In our study, the fat phase will consist of a complex mixture of lipid fractions, including rapeseed oil, two distinct fractions of milk fat, and emulsifiers, to closely replicate the lipid composition of “spreadable fat”, typically emulsified with an aqueous phase [10]. The choice of rapeseed oil provides a nutritional balance in terms of the unsaturated fatty acid/saturated fatty acid ratio, as well as very low atherogenicity (AI) and thrombogenicity (TI) indices [11]. Several recent studies, though still limited in number, use AMF as a specific fat to compensate for the fluidity provided by olive, corn, and flax oils [12]. In our case, we studied a more complex mixture of specific fats, namely a blend of AMF and AMF stearin, which increases the solid fat content (SFC) and texture. This approach has already been implemented by Li et al. [13] to limit the impact of oxidation reactions of polyunsaturated oils mixed with milk stearin. However, all these formula modifications lead to changes in SFC, nucleation, and crystallization kinetics.

Many works study the cooling kinetics of fats strongly influencing the appearance of polymorphic forms (unstable hexagonal form α, the metastable orthorhombic form β′ and the stable triclinic form β) [14,15,16]. Although the crystallization of dairy fats is widely studied [17], few studies focus on the crystallization of complex mixtures of dairy fats and vegetable oils. One notable study is by Kaufmann et al., which examines the crystallization properties of a binary mixture consisting of anhydrous milk fat (AMF) and rapeseed oil (RO). The authors show that the addition of vegetable oil modifies the melting characteristics, microstructure, texture, and crystallization kinetics of AMF [10].

The objective of our study is to analyze, through different multiscale approaches (DSC and X-ray diffraction techniques), the crystallization behavior, in term of crystal types and forms (polarized microscopy) and melting resistance (calculation of SFC), at 4 °C of fats derived from a mixture of polyunsaturated oil (rapeseed oil, RO) and anhydrous milk fats (AMF and AMF41), in the presence of emulsifiers such as mono- and diacylglycerols, which are widely used in the industry [18,19,20,21,22]. The impact of added emulsifiers is also studied in terms of their role as crystallization initiators, a topic that has been mentioned in very few studies.

## 2. Materials and Methods

### 2.1. Materials

Industrial samples of anhydrous milk fat (AMF) and stearin of AMF (AMF41) were supplied by S.A. Corman (Limbourg, Belgium). The mono- and diacylglycerols (PS) ingredient GRINDSTED^®^ PS 209 and monoglycerides (DMG) ingredient Palsgaard^®^ DMG 0291 were purchased from DANISCO S.A.S (Neuilly-sur-Seine, France) and Palsgaard A/S (Juelsminde, Denmark) respectively. The rapeseed oil (RO) comes from a local French producer. All LC/MS grade solvents (acetone, acetonitrile, hexane, chloroform, methanol, diethyl ether) and formic acid were purchased from Sigma-Aldrich (St. Louis, MO, USA). Fatty acids PUFA standards (Grain FAME Mix, PUFA No.2 Animal Source, Supelco, Bellefonte, PA, USA), triacylglycerol standards triolein (OOO), and tristearate (SSS) were purchased from Sigma-Aldrich (St. Louis, MO, USA). Other triacylglycerol standards 1-palmitoyl-2-oleoyl-3-stearoyl-rac-glycerol (POS), 1,3-palmitoyl-2-oleoyl glycerol (POP), and 1,3-distearoyl-2-oleoyl glycerol (SOS) (Cayman Chemical Company, Ann Arbor, MI, USA) were acquired from Bertin Bioreagent (Montigny-le-Bretonneux, France).

### 2.2. Blend Preparation

A typical blend was prepared in a weight ratio with RO (58.28%), AMF (27.68%), AMF41 (12.08%), DMG (1.36%), and PS (0.58%). All ingredients were mixed in a thermostatic reactor at 80 °C under 250 rpm for 30 min, stirring until a homogenous fat blend was obtained. The mixtures are studied at different temperatures of 4 °C, 10 °C, and 35 °C, which correspond, respectively, to refrigerator exit temperature, spreadable fat usage temperature, and tasting temperature to detect any potential textural and organoleptic defects.

### 2.3. Chemical Characterization

#### 2.3.1. Lipid Classes Analyses

Neutral and polar lipids quantification were obtained by Iatroscan MK-5 thin-layer chromatography—flame ionization detection (TLC-FID) (Iatron Laboratories Inc., Tokyo, Japan). Each sample was diluted in chloroform/methanol 2:1 (*v*/*v*) at 10 mg mL^−1^ and spotted on Chromarod S-III silica coated quartz rods held in a frame. The migration was conducted for 20 min in a solution of hexane/diethyl ether/formic acid 80:20:0.2 (*v*/*v*/*v*), afterwards oven dried for 1 min at 100 °C, and finally scanned by the Iatroscan analyzer. The following measurement conditions were set at 160 mL min^−1^ for the hydrogen flow rate and at 2 mL min^−1^ for the air flow rate. Peak recording and integration were performed with ChromStar^®^ software V4.14 (SCPA, Weyhe-Leeste, Germany). Samples were analyzed in triplicate. Triacylglycerols (TAG), diacylglycerols (DAG), and monoacylglycerols (MAG) standards were used to perform the calibration range.

#### 2.3.2. Fatty Acids Quantification

Fatty acids (FA) profiles of the samples were determined by gas chromatography—flame ionization detection (GC-FID). Fatty acid methyl esters (FAMEs) were prepared according to the Ackman method [23]. Separation and quantification of FAMEs were performed by a Shimadzu GC-2010 (Kyoto, Japan), equipped with a flame-ionization detector and a fused silica capillary GC column (60 m × 0.25 mm × 0.20 μm, SPTM2380, Supelco, Bellefonte, PA, USA). Injector and detector temperatures were kept at 250 °C. The column oven temperature was programmed as follows: initially set at 120 °C for 2 min, followed by a ramp to 180 °C at a heating rate of 40 °C min^−1^, then maintained at 180 °C for 2 min. Next, the temperature was increased to 220 °C at a heating rate of 3 °C min^−1^, and finally, a 25-min isotherm was applied. The data were performed with manufacturer “GC solution” software V2.41.00. Fatty acids were determined by comparison of their retention times with standard mixtures from vegetal and animal sources (Grain FAME Mix, PUFA No. 2 Animal Source, Supelco, Bellefonte, PA, USA). All samples were analyzed in triplicate with nonadecanoic acid (C19:0, Sigma-Aldrich, St. Louis, MO, USA) as the internal standard (IS). The fatty acid compositions were used to calculate the nutritional quality through the atherogenicity index (AI) and thrombogenicity index (TI), in accordance with Silva et al. [4].

#### 2.3.3. Triacylglycerols Determination

The analysis was conducted using a Shimadzu reverse-phase high-performance liquid chromatography (HPLC) system (Shimadzu, Kyoto, Japan) equipped with an auto-injector (SIL-20AC HT), a pump (LC-201D), a column oven (CTO-20AC), a refractive index detector (RID-10A), a system controller (CBM-20A), and “LC solution” software V1.25 (Shimadzu, Kyoto, Japan). Samples were prepared in a 5% chloroform solution and subsequently injected on reverse-phase C18 column (150 mm × 4.6 mm, 5 µm particle size, Apollo, Grace, Belgium). The mobile phase was acetone and acetonitrile in a 50:50 (*v*/*v*). The oven temperature was maintained at 30 °C, and a flow rate of 1.2 mL min^−1^ was employed during the 60-min analysis. The analysis was performed in triplicate. The triacylglycerols (TAG) method identification uses the equivalent carbon number (ECN) described by the formula ECN = CN − 2n, where CN and 2n represent the carbon number and the number of double bonds, respectively, according to [24]. Triacylglycerols OOO, SSS (Sigma-Aldrich, St. Louis, MO, USA), POP, SOS, and POS (Cayman Chemical Company, Ann Arbor, MI, USA) were used as standards for calibration.

### 2.4. Polarized Light Microscopy

The samples are heated for 30 min at 80 °C and then cooled by the Peltier effect to 4 °C at different speeds: 1 °C min^−1^, 5 °C min^−1^, and 10 °C min^−1^. Samples were observed after stabilization at 4 °C using an Olympus (Provis AX70, Tokyo, Japan) polarizing optical microscope equipped with a halogen lamp supply unit Olympus TH4-200 and a grey-level microscope charge-coupled device (CCD) camera.

### 2.5. Determination of Solid Fat Content by Pulsed Nuclear Magnetic Resonance (p-NMR)

Solid fat content (SFC) measurement of samples was performed using a pulsed nuclear magnetic resonance (NMR) spectrometer (Minispec-mq20, Bruker, Karlsruhe, Germany) and based on the indirect method according to ISO 8292-2:2010. The SFC values were determined at temperatures of 10, 20, 30, and 35 °C [25]. Samples were analyzed in triplicate.

### 2.6. Thermodynamic Analysis

The thermal behavior of samples was determined by Differential Scanning Calorimetry (DSC) using TA Discovery DSC 2500 (TA instruments, New Castles, DE, USA), equipped with a refrigerated cooling system RCS120. The DSC instrument was enthalpy-calibrated with indium with a melting temperature of 156.60 °C. Samples (approximately 10 mg) were sealed in aluminum Tzero hermetic pans, and an empty pan was used as a reference. The system was purged using nitrogen. The results were performed in triplicate and analyzed with Trios Software V5.0 (TA Instruments, New Castles, DE, USA).

#### 2.6.1. Non-Isothermal Differential Scanning Calorimetry (DSC) Method

The non-isothermal method protocol used: an isothermal for 30 min at 80 °C to melt and erase the crystal history of the sample, then cooled at a rate of 5 °C min^−1^ to −80 °C, afterward with an isothermal for 1 min and a heating step to 80 °C at 5 °C min^−1^. SFC profiles of the sample were determined by melting profiles that were obtained using the same program with a temperature ramp at a rate of 5 °C min^−1^ [23].

#### 2.6.2. Isothermal DSC Method

For the isothermal DSC analysis using the stop-and-return method [26], the following time-temperature program was implemented: isothermal for 30 min at 80 °C to fully melt the sample and erase its crystal history; cooling to isothermal temperature at 4 °C with a cooling rate of 5 °C min^−1^ for different times (2, 5, 16, 32, 60, 120, and 180 min); heating at a rate of 5 °C min^−1^ to 80 °C and maintaining this temperature for 30 min.

#### 2.6.3. Avrami Theory Calculation

The Avrami macrokinetic model is the most commonly approach used to describe isothermal crystallization kinetics [27,28,29]. The relative degree of crystallinity, denoted as X(t), is consequently associated with the crystallization time, t, in accordance with Equation (1):

(1) $$X\left(t\right)=1-\exp\left(-kt^{n}\right)$$

where, n represents the Avrami exponent, a variable tied to the nucleation process, and k denotes the growth function, dependent on both nucleation and crystal growth, t as time variable, and X(t) the crystallized fraction as function of time. The parameters k and n depend on the temperature. Adjustments with the least squares method were carried out on the 180-min isotherm at 4 °C when an exothermic peak was observed.

### 2.7. DSC-Raman Spectroscopy

Thermal heating measurements were carried out using a TA Q200 (TA instruments, New Castles, DE, USA) with an in-situ Raman spectroscopy coupling system. The spectrometer Kaiser RXN1 (Kaiser Optical Systems Inc., Ann Arbor, MI, USA) was employed with a 400 mW power and a laser wavelength of λ = 785 nm. The time-temperature program used is as follows: heating to 80 °C for 30 min and decrease to 4 °C at a rate of 5 °C min^−1^, isothermal for 1 min, then increase temperature at 80 °C at a rate of 5 °C min^−1^. The acquisition time for each spectrum was 5 s every 3.82 °C with an acquisition range of 0 to 3460 cm^−1^. Baseline correction and data normalization of the melt and crystallized spectra were carried out using a program with MATLAB^®^ R2023b V23.2 software (The MathWorks, Inc., Natick, MA, USA) based on Bouita’s work [30,31]. The spectra were post-processed by applying a Savitzky–Golay filter, followed by a baseline subtraction using the following point at 678, 755, 1233, 1574, and 1779 cm^−1^ [32]. The normalization of the spectra was performed based on the total area. Signals within the range of 700 to 1800 cm^−1^ are displayed.

### 2.8. X-ray Diffraction Spectroscopy (XRD)

The fat crystal polymorphic forms of samples were determined by X-ray diffraction (XRD). First, all were heated at 80 °C for 30 min in order to melt all existing crystals, then the samples were cooled at 4 °C with a rate of 5 °C min^−1^. Multiple XRD measurements were realized at 4 °C for 180 min in order to follow any structural changes, and then were summed together by groups of five spectrums, if no modification was observed. The XRD measurements were performed using a Panalytical X’Pert Pro diffractometer (Malvern Panalytical B.V., Almelo, NL, USA) equipped with a Cu anode X-ray tube and a Ge (111) incident-beam monochromator (λ = 1.5406 Å) to exclude the Kα2 line; the low temperature (4 °C) was reached within a Phenix cryostat (Malvern Panalytical B.V., Almelo, NL, USA) under a primary vacuum (~1 mBar). The X’Celerator detector (Malvern Panalytical B.V., Almelo, NL, USA) which functioned as a “scanning line detector (1D)” with a 2.122° (2θ°) active length. Data collection was conducted within the scattering angle range of 2–50° using a 0.0167° step over 180 min. Low-angle X-ray diffraction data, from 1° to 10°, were measured after the 180-min isotherm by changing the optics geometry [33].

### 2.9. Statistical Analysis

A two-way ANOVA was used to determine significant differences in the SFC measurements. The analysis was conducted at a significance level of 0.05 to assess significant differences in the same sample at different successive temperatures, as well as for a given temperature between AMF and AMF41, and between AMF and the blend.

## 3. Results and Discussion

### 3.1. Lipid Classes and Fatty Acids Composition

The objective of this study is to investigate the crystallization of a mixture of oil and fats at a temperature of 4 °C. The composition of the blend formulation is based on preliminary results from a mixture design and comprises a mixture of rapeseed oil (RO), anhydrous milk fat (AMF and AMF41) and mono- and diglyceride emulsifiers (E471; PS and DMG) commonly used in industry to initiate and accelerate crystallization [20]. This blend has been selected to obtain the desired taste and texture characteristics. Emulsifiers are also used to stabilize emulsions at O/W or W/O interfaces [34]. The overall lipid class composition of the blend shows 87.4 ± 2.4% TAG, 0.4 ± 0.1% DAG, 1.1 ± 1.1% MAG, and 11.1 ± 3.4% polar lipids. The TAG fraction comes exclusively from rapeseed oil (97.7 ± 0.8%) [35] and anhydrous milk fat (86.5 ± 3.1% and 84.6 ± 0.5% in AMF and AMF41, respectively). We also note the presence of complex polar lipids [36] (glycerophospholipids and sphingophospholipids) at 13.0 ± 3.2% and 14.6 ± 0.6% in AMF and AMF41, respectively, in accordance with the study of Bourlieu et al. [37], as well as the minority presence of DAG and MAG, such as that reported by Vanhoutte et al. [38]. The surfactants used were DMG and PS with 1.36% and 0.58% of the fatty phase, respectively. These were mainly characterized by 97.7 ± 0.8% and 85.1 ± 0.9% MAG and 2.3 ± 0.8% and 1.9 ± 0.4% DAG, respectively, for DMG and PS. The total emulsifiers used (1.9% of blend) represents, for example, only 1.5% of a blended spread product containing 80% fat, knowing that this type of ingredient is tolerated in sufficient quantity (quantum satis) [1].

The use of anhydrous milk fat adds texture through the presence of saturated fatty acids (SFA) (Table 1), 62.05% (AMF), and 71.81% (AMF41) through the presence of short-chain esterified FA, commonly accepted to be responsible for the butter flavor [8,39,40]. The presence of esterified SFA is necessary for the texturization of semi-crystalline matrices and does not influence lipid metabolism. For example, short-chain fatty acids (C4:0, C6:0), medium-chain fatty acids (C8:0, C10:0), and stearic acid (C18:0) have no proven deleterious effects and are necessary for metabolism as confirmed by European Food Safety Authority (EFSA, [41]) and L’Agence nationale de sécurité sanitaire de l’alimentation, de l’environnement et du travail (ANSES, [42]). However, some are said to be atherogenic (lauric, myristic, and palmitic acids) and may present deleterious effects when in excess. The combination of vegetable oil and fat of animal origin [1] provides a wide range of fatty acids, such as MUFA, which are widely present in rapeseed oil (C18:1 n-9, 61.36%), and PUFA (C18:3 n-3, 7.20%; C18:2 n-6, 19.19%). These PUFAs, known as essential fatty acids, namely linoleic acid and alpha-linolenic acid, are precursors for the synthesis of other fatty acids necessary for proper metabolism. Their presence remains a beneficial asset for health [43,44,45]. ANSES recommends a dietary intake of 4% and 1% of the daily energy intake, respectively, for linoleic acid (LA) and alpha-linolenic acid (ALA) [46]. Within the population, the recommended dietary intake for ALA are not met unlike LA. ANSES recommends a LA/ALA ratio of less than 5 to ensure a satisfactory nutritional quality diet, which amounts to 2.40 for the formulated blend [47].

The atherogenic (AI) and thrombogenic (TI) indices, which are directly related to the health of lipid bases and take into account the effects of saturated FA and unsaturated FA, are calculated from the fatty acid composition table (Table 1). For both parameters, AMF showed the highest values, with 11.83 (AI) and 10.60 (TI) and 11.48 and 20.04 for AMF41, respectively. Rapeseed oil was chosen for its very low indices, which are consistent with the literature: 0.09 (AI) and 0.19 (TI) [12]. The addition of a significant amount of rapeseed oil (58%) significantly reduces the indices of the blend to 1.13 (AI) and 0.90 (TI) compared to the dairy fat used. These values are consistent with the margarine indices in the Da Silva review [4].

### 3.2. Triacylglycerol Composition

In the rapeseed oil used, triunsaturated TAGs are mainly found, i.e., OOO (29.38%), LOO (22.51%), LnOO (14.12%), in accordance with the work of Neff et al. [48] and Ravotti et al. [49]. The dairy fats used contain a wide variety of TAG, 200 of which have been identified. The majority are asymmetrical TAG (saturated-unsaturated-unsaturated (SUU) or saturated-saturated-unsaturated (SSU)) with a saturated or unsaturated FA in position *sn-1* or *sn-3* [50]. The TAG of AMF and AMF41 proposed in Table 2 are in agreement with the literature [51,52,53]. Anhydrous milk fat stearin contains higher levels of trisaturated TAG with long-chain fatty acids (MPP, MSS), triunsaturated TAG such as triolein (6.75%), and asymmetrical TAG with long- and medium-chain FA (POP, MPO, SOO) than AMF, which is richer in short-chain TAG such as butyric acid (Bu, C4:0), caproic acid (Co, C6:0), and caprylic acid (Cy, C8:0), as reported in different works [8,16]. The FA and TAG compositions of milk fat vary according to season and diet, as well as fractionation processes [16,51]. The high content of esterified oleic acid in POO (4.67% and 3.48% AMF and AMF41) and MOO (2.82% and 5.85% AMF and AMF41) indicates summer milk fat, while the capric acid (C, C10:0) and myristic acid (M, C14:0) contents (Table 1) are representative of summer butterfat [51]. The composition of the blend features a diversity of TAG from both oil and fat. There is a large number of asymmetrical TAGs provided by dairy fats. In a complex mixture, asymmetric TAGs generally tend to form a β′ structure, while symmetric TAGs tend to form a β structure. The addition of rapeseed oil enriches the blend in triunsaturated TAGs compared to dairy fats. The increase in the triolein (OOO) fraction facilitates polymorphic transitions, leading to more stable polymorphic forms [54].

### 3.3. Effect of Cooling Rate on Blend Constituents Observed by Polarized Light Microscope

**Figure 1:**
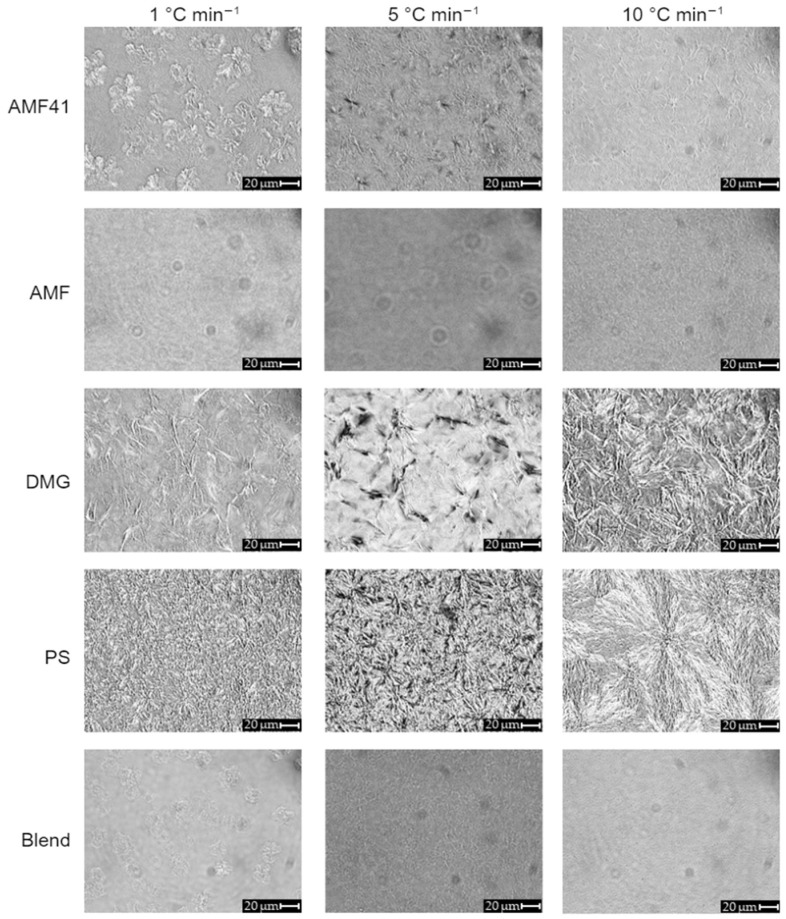
Microstructure of anhydrous milk fat (AMF and AMF41), mono- and diacylglycerols (PS and DMG) and blend (RO (58%), AMF (28%), AMF41 (12%), DMG (1.4%), and PS (0.6%)), acquired under polarized light at 4 °C after different cooling rate ramps (×50).

Polarized light microscopy allows us to observe the organization of triacylglycerol mixture crystals, which crystallize as nanoplatelet aggregates. The results (Figure 1), imaged at 4 °C, show that cooling kinetics induce different crystal shapes and sizes (needles, spherulites). These shapes are not correlated with their polymorphism [9,51,55]. Cooling rates influence the nucleation step of crystallization, which plays a crucial role in the number and size of the crystals formed, their polymorphic form, and the final distribution of the crystalline solids. A slow cooling rate allows more time for the crystals to arrange themselves more uniformly [55,56].

Three cooling rates were studied up to 4 °C (1, 5 and 10 °C min^−1^). TAG crystals appear white, while the liquid TAG fraction appears black in polarized microscopy photographs. At high kinetics (10 °C min^−1^), the crystals are smaller and less ordered than the large crystals obtained via slow cooling kinetics (1 °C min^−1^). These results are in line with microscopic observations found in the literature [50,57]. Spherulites, consisting of a central core and a radial lamellar stack of crystals (white), were observed only at a 10 °C min^−1^ cooling rate for the PS emulsifier [58]. An arrangement in the form of very fine needles is observed for PS and DMG at 1 °C min^−1^ kinetics, this form being typical of crystals in β′ form [16]. Cooling AMF to 4 °C with slow kinetics (1 °C min^−1^) results in the formation of spherulite-type crystals observable also for AMF41 and blend. Due to its richer composition in triacylglycerol fraction crystallizable at 4 °C, AMF41 presents larger and more numerous crystals than AMF and blend. During rapid cooling, granular microstructures are composed of a large number of small crystals within AMF, AMF41, and blend. Crystallization proceeds more rapidly and nucleation events predominate over crystal growth processes [15,50,59]. The blend of oil and fats shows less clear-cut and diffuse structures in terms of crystalline microstructures, given the richness of the non-crystallizable TAG fraction at 4 °C, regardless of kinetics.

### 3.4. Thermal Behavior by DSC

In Table 3, only peaks with the maximum enthalpy of melting and crystallization peaks are shown. The curves for AMF, AMF41, and blend show both endothermic and exothermic peaks. According to several studies, the low-melting triacylglycerols (LMP) fraction is characterized by melting temperatures inferior to 10 °C, the medium-melting triacylglycerols (MMP) fraction melts between 10 °C and 20 °C, and the high-melting triacylglycerols (HMP) fraction melts at temperatures above 20 °C [50,60,61]. These different fractions proportions are dependent on the triacylglycerols’ content. The melting curve recorded by DSC during the heating of AMF shows these three typical endothermic peaks, corresponding to the low melting point (LMP) fraction from −23.80 °C to −5.37 °C with a peak temperature of −2.97 °C, medium (MMP) fraction from −5.37 °C to 21.38 °C with a peak temperature of 12.60 °C, and high (HMP) from 21.38 °C to 33.59 °C with a peak temperature of 32.33 °C. In a study of Lopez and Ollivon [60], the three fractions delimited in temperature during the heating of AMF are the LMP fraction from −40 °C to 8 °C, the MMP fraction from 8 °C to 22 °C, and the HMP fraction from 22 °C to 37.5 °C. These peaks are found within dairy stearin (AMF41) with fractions delimited by the following endothermic temperatures: the LMP fraction from −12.98 °C to 9.68 °C with a peak temperature of 6.75 °C, the MMP fraction from 9.68 °C to 18.30 °C with a peak temperature of 13.58 °C, and the HPM fraction from 18.30 °C to 44.74 °C, with a peak temperature of 38.57 °C. The LMP fraction of AMF is rich in TAG, containing long-chain PUFA and short-chain SFA, such as BuOO, BuPO, and PPO. The MMP fraction mainly contains TAG made up of short-chain FA or PUFA, such as BuPP and PPO. The MMP fraction is sought after for its organoleptic characteristics, such as its texture and flavor profile, which enhance the sensory qualities of dairy products [50]. The HMP fraction is characterized by a high concentration of long-chain SFA, such as PPP and PSS [14,50].

The presence of rapeseed oil (58.28%) mixed with anhydrous milk fats (AMF and AMF41; 39.76%) results in a slight decrease in endothermic temperature values due to the enrichment of PUFA provided by vegetable oil. A decrease in the LMP fraction temperatures is observed, delimited by the following endothermic temperatures: LMP from −13.66 °C to −1.70 °C with a peak temperature of −6.20 °C, MMP fraction from −1.70 °C to 19.24 °C with a peak temperature of 6.27 °C, and HPM fraction from 19.24 °C to 36.08 °C with a peak melting temperature of 27.47 °C. An additional fraction corresponding to the “very low melting point” TAG (VLMP) of rapeseed oil appears. VLMP fraction from −38.28 °C to −13.66 °C with a peak melting temperature of −19.23 °C, which is similar to the endothermic melting point of rapeseed oil (−18.87 °C). When the blend is stored at 4 °C, part of the TAG content remains liquid (VLMP and part of the MMP fractions). This corresponds to exothermic temperature peaks of −54.99 °C and −57.88 °C for the rapeseed oil and blend, respectively. The balance between the liquid and solid TAGs affects the textural and rheological attributes of the product [62].

#### Solid Fat Content Measurements

SFC values are obtained by both p-NMR and DSC, which are complementary techniques widely used in research fields. It is important to note that DSC is a “dynamic” method which performs measurements at variable temperatures, whereas NMR is a “static” method which maintains the sample at a constant temperature. NMR is a standardized methodology typically used in industry. The differences in values between the two techniques are related to differences in the sample weight and implementation times, which directly influence the results. Additionally, a thermodynamic factor plays a role: the energy consumed per unit of melted mass tends to increase with the melting temperature of each fat fraction, as the melting of TAGs and polymorphic transitions are measured together [63]. The temperatures chosen (10, 20, 30, and 35 °C) for the SFC measurements represent typical industrial melting temperatures and reflect the different usage states of spreadable products, from the fridge to the mouth, in accordance with ISO standards [25].

In Table 4, where the results are compiled, SFC values obtained by DSC are generally higher than those measured by p-NMR [64]. The trends observed with p-NMR and DSC are consistent, which supports the DSC results regarding the SFC. Rapeseed oil has no SFC value between 10 °C and 35 °C due to its molten state, unlike PS and DMG, which have a high melting point and a nearly fully crystallizable fraction at 10 °C, with SFC values approaching 100% and 50% at 10 °C, respectively. SFC is a parameter widely used by the butter and margarine industries. This parameter is linked to the rheological and textural properties of butters and margarines. In particular, for adequate spreadability of a spread, the SFC must be between 7% and 13% at 10 °C, in order to obtain the desired semi-crystalline structure. The product must not exceed an SFC value of 32% at 10 °C. For a temperature of around 20 °C, the SFC value should not be less than 10%, enabling good texturing and limiting exudation from the aqueous phase [4,65,66]. The value of 42.40% obtained for AMF by p-NMR is in agreement with Smet (~40% at 10 °C) [16,61,67]. Within dairy fats at 4 °C, the so-called solid crystallizable fraction is made up of MMP and HMP TAG fraction [50]. Blend shows SFC values measured by DSC similar to AMF at different temperatures, whereas by p-NMR only the value at 35 °C is identical. As shown in Table 4, the SFC values by DSC show that at 20 °C, 30 °C, and 35 °C, there are no significant differences between AMF and the blend. These values are significantly different from the NMR measurements; however, they remain very close. This similarity in SFC suggests that the blend’s formulation effectively mimics the textural and structural properties of AMF.

### 3.5. Raman Spectroscopy

**Figure 2:**
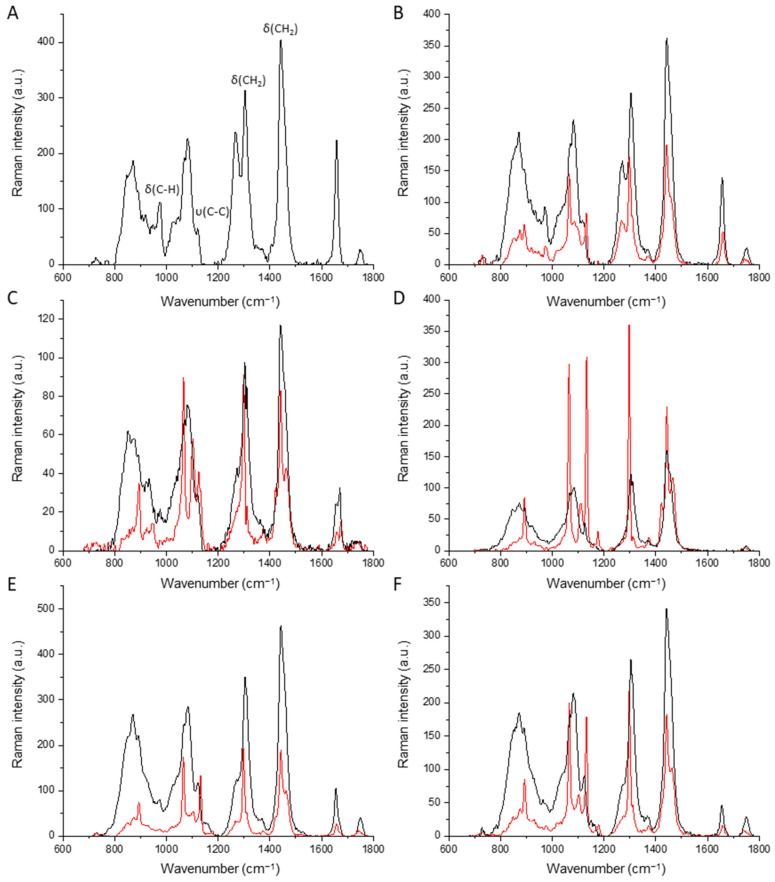
Raman spectra (700–1800 cm^−1^) of melted (black curve) and crystallized (red curve) after removing baseline and normalization correction of RO (**A**), blend (**B**), DMG (**C**), PS (**D**), AMF (**E**) and AMF41 (**F**). Blend: RO (58%), AMF (28%), AMF41 (12%), DMG (1.4%), and PS (0.6%).

Raman spectroscopy is a vibrational spectroscopy method in the infrared that can be used to analyze the composition of fats and oils. This technique is widely used in quality control (e.g., authentication, oxidation, free FA content) and in the determination of lipid classes, oils, and fats [68,69]. Figure 2 shows normalized Raman spectra at 80 °C (black curve) and 4 °C (red curve) of RO (A), DMG (C), PS (D), AMF (E), AMF41 (D), and blend (B). The profiles are nearly identical, given the broad spectrum of fatty acids from the oil, fats, and emulsifiers in the blend. The positions of the Raman peaks of the oil and fats are similar due to the fact that they are essentially composed of TAG, saturated and unsaturated FA with aliphatic chain lengths from C4 to C24.

The primary Raman peak positions and the respective vibrational modes of oil, fat, and surfactants are shown in Table 5 follows: 1746 cm^−1^ υ(C=O) carbonyl stretching from ester, 1659 cm^−1^ υ(C=C) olefinic stretching from unsaturated bonds of *cis* conformers, 1442 cm^−1^ δ(CH2)sc bending (scissoring) from methylene, 1298 cm^−1^ δ(CH2)tw bending (twisting) from methylene, 1267 cm^−1^ δ(=CH)ip in-plane olefinic hydrogen deformation from unconjugated *cis*, 1131 cm^−1^ υ(C-C)ip stretching from methylene with in-phase aliphatic stretch all-*trans*, 1083 cm^−1^ υ(C-C)g stretching from methylene with in-phase aliphatic in gauche, 1065 cm^−1^ υ(C-C)op stretching from methylene with out-of-phase aliphatic all-*trans*, 971 cm^−1^ δ(C=C) bending (deformation) from *trans* of the carbon-carbon double bond and 869 cm^−1^ υ(C–C) stretching from methylene [68,70,71,72,73]. The bands corresponding to unsaturation (971, 1267 and 1659 cm^−1^) are more intense in rapeseed oil, used in this blend to provide essential FA (LA, ALA) [68,70,71]. The PS emulsifier shows strong intensities at 1442 and 1298 cm^−1^, corresponding to the presence of saturated aliphatic chains. No signal is observed at 1659 cm^−1^ υ(C=C) stretching from unsaturated banding. The intensity of the bands (C=C) corresponding to aliphatic chains is higher in the AMF41 fraction, in line with the fatty acid composition table (Table 1). The AMF41 stearin has 71.81% SFA, compared to AMF 62.05% with a lower melting point (Table 3). These results are in accord with the data of Gómez and Mascaraque [74]. Double bond configurations of the *cis* aliphatic chain are predominant in all samples. However, a slight peak is observed around (DMG, AMF, and AMF41) 1670 cm^−1^ corresponding to υ(C=C) stretch from unsaturated banding *trans*, in agreement with the literature [73,74]. The band near 1746 cm^−1^, corresponding to the relatively weak stretching of the carbonyl ester bond υ(C=O), is present in all melt and crystallized spectra (Figure 2D) [68,71]. The highest intensities of this band are found for blend, AMF, and AMF41 due to the richness of various short-chain SFA present within these materials [68].

### 3.6. Effect of Isothermal Temperature (4 °C) on Crystallization Behavior of Blend and Constituents

**Figure 3:**
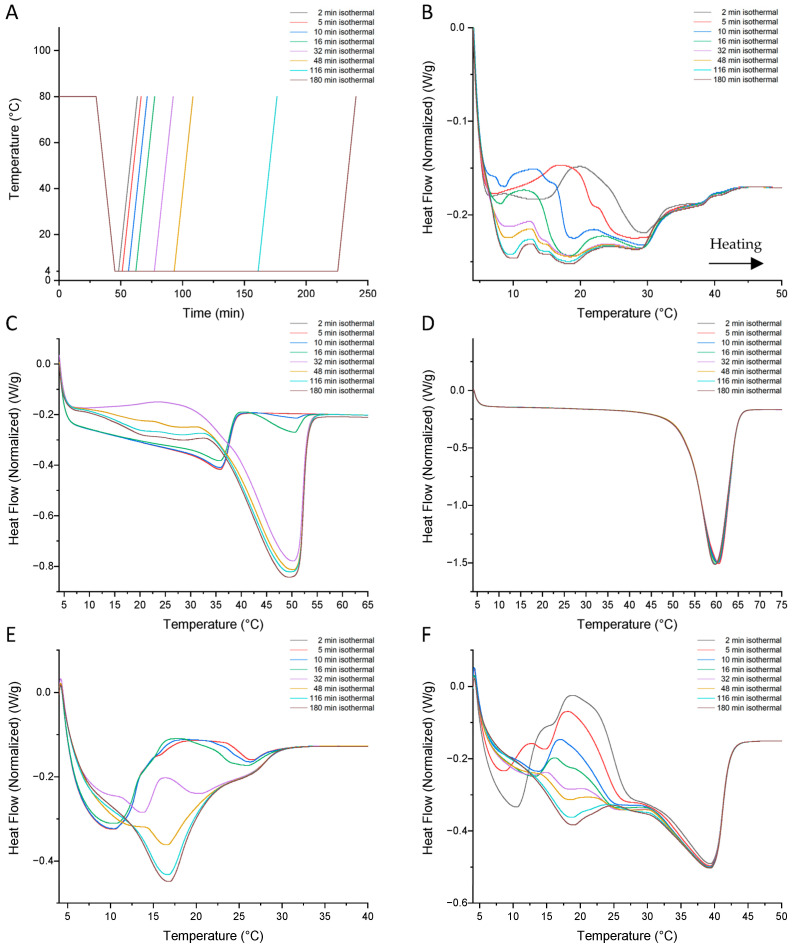
Time-temperature DSC program (**A**) and endothermic profiles obtained via stop-and-return method with a rate of 5 °C min^−1^ and different crystallization isotherm times at 4 °C for blend (**B**), DMG (**C**), PS (**D**), AMF (**E**), and AMF41 (**F**).

The stop-and-return method was developed by Foubert et al. [26] to observe the polymorphism behavior and to bring information about the two-step crystallization of lipids [10,26,57]. The use of stop-and-return allows us to study the crystallization behavior and polymorphic transition rate of a fat and oils blend at 4 °C. During the heating step, an endothermic peak corresponding to the melting of the crystals formed during the cooling and isothermal step is observed [75]. Figure 3A shows the isothermal (time–temperature) program applied, in line with the work of Wiking’s team [57]. In Figure 3, a shift in endothermic peaks towards higher temperatures indicates a transition to a more stable polymorph. When the area of an endothermic peak increases at the same temperature, this means that the polymorphic fraction continues to crystallize. The first stage of crystallization is a metastable polymorphic α form, which continues towards a more stable β′ form. This polymorphic transition is observable by an exothermic phenomenon (Figure 3B,C,E,F) [57,76]. In Figure 3, from a 2-min isotherm at 4 °C, an endothermic peak appears with a melting temperature around 10 °C for the blend, AMF, and AMF41 (B, E, and F), representing the first α crystals. In Figure 3E, the thermogram obtained for AMF shows an endothermic peak (10 °C) that disappears between the 16- and 32-min isotherm. At around 18 °C, an exothermic peak shows the polymorphic transition of α crystals into β′, up to the 32-min isotherm. This observation is in agreement with Wiking’s results, who declares the second crystallization step observed by DSC at 18.5 min, which was confirmed by X-ray diffraction to an α to β′ phase transformation [57]. The transition is relatively quicker for AMF41, with the first endothermic peak (10 °C) disappearing after 10 min of isothermal time, and the exothermic peak at around 18 °C appearing until 16 min isothermal, when it disappears between 16 min and 32 min of isothermal time (Figure 3F). Comparing the profiles of AMF and AMF41 during the first minutes of the isotherm, the endothermic peak representing β′ crystals is much important at 4 °C, due to the enrichment of MMP and HMP fractions in anhydrous milk fat stearin. The exothermic peak (10 °C) disappears after 32 min of isothermal for AMF and 16 min for AMF41 and blend. These fat crystals develop not only from HMP in milk fat, but also from the MMP fraction with a carbon number of C36 to C42, according to the work of Danthine or Nguyen et al. [40,76]. Due to the co-crystallization of the different TAG molecule fractions in milk fat, a slower reorganization of the crystal lattices is observed. Nguyen’s results show unfinished polymorphic stabilization after 120 min at 12 °C for AMF [76]. In our case, the results show crystallization tending to reach equilibrium after the 116-min isotherm at 4 °C for AMF and AMF41. Unlike the emulsifiers, no polymorphic transition and crystalline change is visible for PS, which is already stabilized in its polymorphic form within the first few minutes. A transition of α crystals to a more stable form is visible as early as a 10-min isotherm with equilibrium reached after the 32-min isotherm for DMG. Blend shows similar crystal behavior to anhydrous milk fats, reaching equilibrium after 116 min at 4 °C. However, the polymorphic transition is initiated more rapidly. The fraction represented by the endothermic peak temperature (10 °C) is higher in the blend due to the TAG contributed by rapeseed oil. The endothermic peak at around 18 °C represents the TAG of rapeseed oil and anhydrous milk fats. Above 32 °C, TAG are provided by stearin (AMF41) and emulsifiers (PS and DMG).

Avrami theory is a well-known method for describing and/or predicting the kinetics of the thermodynamic phase transformation of fat and blend [77,78]. Calculations of Avrami’s k and n coefficients are performed on the 180 min isotherm, where an exothermic peak was observed for the AMF, AMF41, DMG, and blend samples. No exothermic peak is observed for the PS sample, which is already in the form of stable crystals at 4 °C (Figure 3).

According to Table 6, the DMG has an exponent value n equal to 4, which corresponds to heterogeneous nucleation and spherulitic growth from sporadic nuclei in three dimensions, and also suggests that the nucleation rate is constant and independent of time [79,80,81,82]. A recent critical review written by Shirzad and Viney [82] details possible applications of the Avrami equation in life sciences. Under the same conditions of temperature and time, the blend and anhydrous milk fats (AMF and AMF41) have an Avrami coefficient value n~2 at 4 °C during the 180-min isotherm. The value of AMF’s n exponent is 2.30, slightly lower than that of Cooney et al. [59], who found a value of 2.86. This n~2 value corresponds to crystallization that may be disc-like growth from instantaneous nuclei or rod-like growth from sporadic in two dimensions, according to Abu Bakar’s results [83]. This value also reflects a rapid nucleation rate at the start, which attenuates with time [79]. The results suggested that adding a significant amount of rapeseed oil to the blend does not affect the nucleation step, which is accelerated by the presence of emulsifiers. The half-crystallization time (t_1/2_) is linked to the composition of the triacylglycerol fractions. This time is longer when the milk fat contains a higher TAG LMP fraction, with values of 22.58 and 8 min (Table 6), respectively, for AMF and AMF41. Differences in the chemical composition of fats lead to different crystallization mechanisms. Multidimensional crystal growth is favored by the presence of MMP and LMP fractions in milk fat [79]. Studies have shown that the influence of DAG and MAG on the crystallization behavior of milk fat depends on temperature, composition, and their concentrations. MAG with stearic acid (S, C18:0) accelerate TAG crystal growth and the polymorphic transition from α to β′. In contrast, MAG with oleic acid (O, C18:1 n-9) do not affect crystal behavior, while MAG with lauric acid (La, C12:0) show intermediate behavior [50].

### 3.7. Crystal Polymorphism Study

**Figure 4:**
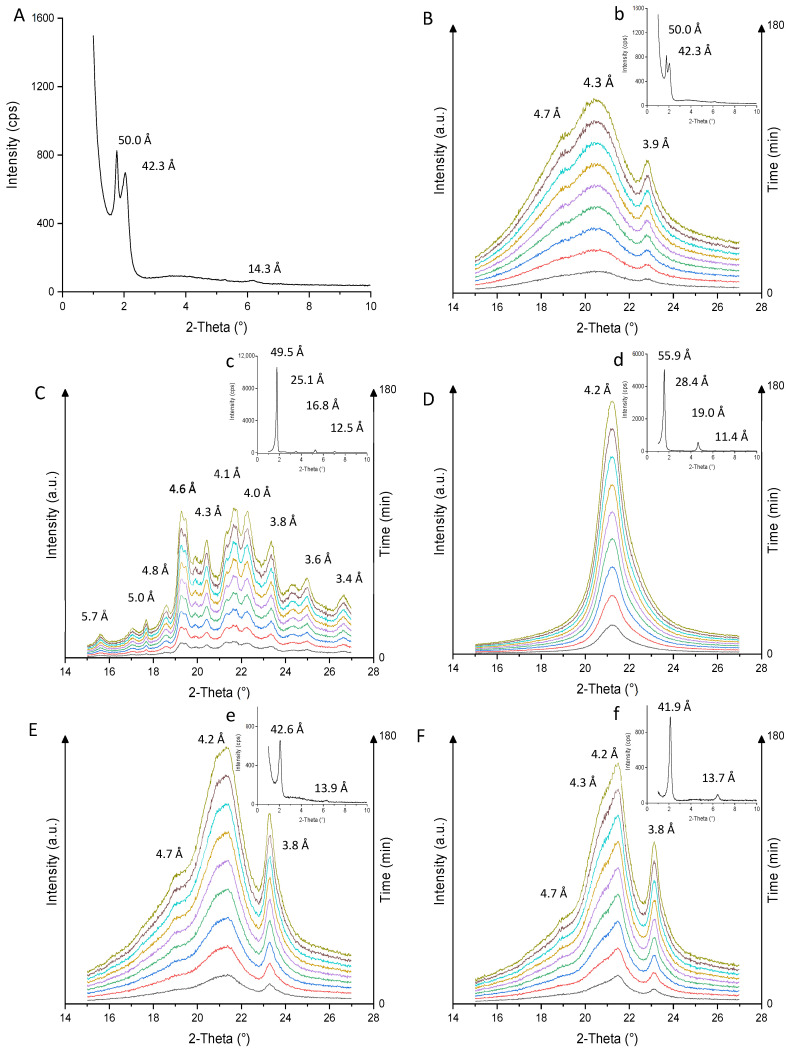
Wide-angle (15–27°, (**B**–**F**)) and low-angle (0–10°, (**b**–**f**), A: zoom of b) XRD diffractograms measures during the 180-min crystallization isotherm at 4 °C for blend ((**A**): zoom of (**B**,**b**)), DMG (**C**,**c**), PS (**D**,**d**), AMF (**E**,**e**), and AMF41 (**F**,**f**). The wide-angle measurement data is accumulated in ascending order according to the isothermal time, ranging from 0 to 180 min. Blend: RO (58%), AMF (28%), AMF41 (12%), DMG (1.4%), and PS (0.6%).

The reticular distances between atom planes characteristic of polymorphic forms (α, β, β′) are determined by wide-angle X-ray diffraction (WAXS). Generally, the β polymorph is not the majority form observed in milk fat and may be present in small quantities during slow cooling. Stable, compact, and coarse β crystals can give rise to an undesirable granular texture in butter, dairy spreads, and blended spreads. Milk fat is a β′-tending fat due to a high diversity of triacylglycerols with a high rate of asymmetric TAG present [40,84], in agreement with the results obtained in Table 2. The β′ crystalline form is ideally sought for good plasticity [10,16,55]. TAG crystallize in lamellar structures composed of two (2L) or three (3L) chain lengths with spacings generally close to 40–50 to 55–70 Å for bilayered (2L) and trilayered (3L) stacking, respectively. These structures are determined by low-angle DRX measurements [10,50]. Figure 4 shows the XRD profiles of crystallization at 4 °C during 180 min isotherm after a cooling kinetic of 5 °C min^−1^. Cumulative diffractograms of wide-angle measurements (15–27°; blend (B), DMG (C), PS (D), AMF (E), and AMF41 (F)) and profiles of low-angle measurements at 180 min (0–10°; blend (a, b), DMG (c), PS (d), AMF (e), and AMF41 (f)) are shown. After 180 min at 4 °C, DMG (Figure 4C), composed of 97.7 ± 0.8% MAG, shows numerous short spacings, predominantly at 4.6, 4.1 and 4.0 Å and of lesser intensity at 4.3 and 3.8 Å, and a 2L-type packing chain length (49.5 Å). These values correspond to a stable polymorphic β-form, typical of unsaturated monoacylglycerol crystals, according to Vereecken’s results [85]. The presence of diffraction bands around 4.2–4.3 Å and 3.8–3.9 Å corresponds to an α-subpolymorph. This sub-polymorphic form is due to the presence of long-chain saturated monoglycerides (i.e., monopalmitin, monostearin, monoarachidonic, and monobehenic), present in the sample according to the FA composition Table 1 [85]. For the PS sample (Figure 4D), a single short spacing (4.2 Å) corresponding to a single α-form is observed, in agreement with the “stop-and-return” results (Figure 4D). The longitudinal arrangement is predominantly under the lamellar trilayer packing 3L structure (55.9 Å) and has an upper arrangement that would appear to be 3L_004_ (19 Å), in agreement with the statements of Lopez et al. [50,86]. Several studies have shown that the addition of MAG and DAG emulsifiers (E471) during the margarine manufacturing process accelerates the crystallization initiation and stimulates the formation of β-shaped crystals [5,6,84]. After 180 min at 4 °C, the blend (Figure 4B), AMF (Figure 4E), and AMF41 (Figure 4F) have similar short spacing, corresponding to polymorph β (4.7 Å), β′ (4.3 and 3.8 Å), and α (4.2 Å). These profiles are similar to those obtained in the literature during an isotherm at 5 °C, with a predominance of the β′ forms over the other coexisting forms [10,61]. The 4.2 Å value corresponding to α -form, converts to β′-form over time, the polymorphic transition from α to β′ form is rapid and is visible by the appearance of the other crystals [10,61]. Polymorphic transition is slowed down when the TAG LMP fraction is high, prioritizing AMF41, AMF, and blend. These results concur with those of the “stop-and-return” results (Figure 3). The profiles of low-angle X-ray measurements after 180 min at 4 °C, blend (Figure 4A), AMF (Figure 4e), and AMF41 (Figure 4f) presenting lamellar structures with a double-chain length organization 2L are 50.0 Å and 42.3 Å (Blend), 42.6 Å (AMF), and 41.9 Å (AMF41). The results for AMF41 agree with those of Lopez and co-workers, who found a value of 41.5 Å for a cooling kinetic of 3 °C min^−1^. She also observes traces of β crystals at low temperatures [50]. The 13.7 Å signal obtained for AMF41 (Figure 4f) could be a 2L1_(003)_ type sheet at 13.7 Å, during a 100-h isotherm at 4 °C. This study concludes the coexistence of 2L (40.5 Å) and 3L (54.2 Å) longitudinal organizations and α, β′2, β′1, and β lateral chain arrangements [86]. Synchrotron radiation X-ray diffraction patterns at wide angles (WAXS) measurements observed the same polymorphic shapes for AMF at low temperatures during slow cooling kinetics (2L1 (41.5 Å) and 2L2 (48.3 Å) sheets) by small angle measurements (SAXS) [50,87]. Our results for the blend sample appear to match the measurements found in these studies (β′-2L (50.0 Å); β′-2L (42.3 Å)), which is a preferable polymorphic form for its organoleptic characteristics in spreadable products’ fat formulations.

## 4. Conclusions

This study focused on the physico-chemical properties and crystallization kinetics at 4 °C of a blend composed of oil, anhydrous milk fats, and emulsifiers. A mixture of oil and dairy fat provides essential fatty acids, particularly from the n-3 series. This supply of long-chain unsaturated fatty acids influences the liquid–solid phase balance within the crystalline network. In the blend, the high content of asymmetric TAGs of dairy origin favors the formation of the β′ crystalline form. The blend has correct SFC values, close to those of milk fat, for use as the fat phase in a spreadable fat (SFC < 32% at 10 °C). The use of the stop-and-return method via DSC allows for the rapid detection and comparison of polymorphic transitions over time at 4 °C. The blend shows similar crystal behavior to anhydrous milk fats, with the polymorphic transition occurring more rapidly. The value of the n exponent in the Avrami equation (n~2) in our study system corresponds to crystallization that may involve disc-like growth from instantaneous nuclei or rod-like growth from sporadic nuclei in two dimensions. These results indicate that it is possible to achieve nucleation similar to that of dairy fats, even in the presence of large amounts of vegetable oil. Polymorphic transition and crystal behavior were studied by DSC and XRD at 4 °C. The predominant polymorphic form of the crystals found in the blend is β′ with an arrangement in 2L form with a crystallization rate of 5 °C min^−1^. The formulation used makes it possible to obtain the ideal crystalline form (β′ > β) required for a spreadable fat and suitable for use as an ingredient in bakery products. The data obtained on the composition and crystalline behavior of the blend (MGC with oil) allow for better control over the development and processing of high-fat products. In perspective, a specific analysis of all the in situ DSC-Raman “spectra” could lead to deepening our knowledge of the crystallinity of the blend and its constituents. Raman spectrometry, already used in quality control and fraud detection [72,88], could contribute to the industrial management of the crystallization of spreadable fat mixtures and the verification of polymorphic forms.

## Figures and Tables

**Table 1:** Fatty acids composition.

Fatty Acids (%)	RO	AMF	AMF41	PS	DMG	Blend
C4:0	-	0.41 ± 0.03	0.42 ± 0.07	-	-	0.14 ± 0.10
C6:0	-	0.94 ± 0.02	0.69 ± 0.02	-	-	0.37 ± 0.13
C10:0	-	2.95 ± 0.04	2.36 ± 0.04	-	-	1.28 ± 0.26
C11:0	-	0.21 ± 0.00	0.20 ± 0.00	-	-	0.10 ± 0.02
C12:0	-	3.81 ± 0.04	3.56 ± 0.04	-	0.17 ± 0.00	1.76 ± 0.26
C14:0	-	10.96 ± 0.09	12.41 ± 0.08	-	0.12 ± 0.00	5.11 ± 0.35
SFA n.d.	-	0.22 ± 0.00	0.20 ± 0.01	-	-	-
C15:0	-	1.03 ± 0.00	1.19 ± 0.01	-	-	0.42 ± 0.12
Iso C16:0	-	0.19 ± 0.00	0.21 ± 0.00	-	-	0.45 ± 0.02
C16:0	4.58 ± 0.04	28.11 ± 0.19	37.48 ± 0.21	4.37 ± 0.01	5.18 ± 0.09	16.21 ± 0.28
C17:0	0.28 ± 0.03	0.75 ± 0.01	0.63 ± 0.01	-	-	0.30 ± 0.01
C18:0	4.01 ± 1.17	11.63 ± 0.06	12.06 ± 0.10	48.68 ± 0.36	-	5.34 ± 0.22
C20:0	0.69 ± 0.03	0.74 ± 0.03	0.35 ± 0.01	7.91 ± 0.05	0.62 ± 0.00	0.13 ± 0.00
C22:0	0.27 ± 0.00	0.10 ± 0.00	0.05 ± 0.00	37.50 ± 0.32	0.36 ± 0.00	0.56 ± 0.10
C24:0	0.13 ± 0.00	-	-	0.84 ± 0.01	-	-
Total SFA	9.96	62.05	71.81	99.3	6.45	32.17
C14:1 n-5 cis	-	1.25 ± 0.01	1.26 ± 0.01	-	-	0.19 ± 0.13
C16:1 n-7 cis	0.22 ± 0.00	0.52 ± 0.01	0.49 ± 0.01	-	0.11 ± 0.00	-
C16:1 n-9 cis	-	1.26 ± 0.01	0.92 ± 0.09	-	0.15 ± 0.00	0.93 ± 0.04
C16:1 isomer n.d.	-	1.01 ± 0.01	0.96 ± 0.08	-	-	-
C17:1 n-7 cis	-	0.43 ± 0.01	0.24 ± 0.03	-	-	0.20 ± 0.02
C18:1 n-5 cis	-	0.10 ± 0.01	0.17 ± 0.07	-	6.65 ± 0.66	-
C18:1 n-7 cis	-	17.00 ± 0.23	6.20 ± 0.04	0.40 ± 0.01	50.19 ± 1.10	-
C18:1 n-9 cis	61.36 ± 1.24	11.26 ± 0.27	14.13 ± 0.36	0.12 ± 0.00	21.20 ± 2.74	49.07 ± 0.84
C18:1 isomer n.d.	-	0.49 ± 0.02	0.41 ± 0.01	-	5.75 ± 1.13	-
C18:1 isomer n.d.	-	0.21 ± 0.01	0.11 ± 0.00	-	1.06 ± 0.03	-
C18:1 isomer n.d.	-	0.34 ± 0.02	0.25 ± 0.01	-	0.75 ± 0.06	-
C18:1 isomer n.d.	-	0.24 ± 0.00	0.25 ± 0.01	-	0.64 ± 0.03	-
C18:1 isomer n.d.	-	-	-	-	0.72 ± 0.02	-
C18:1 isomer n.d.	-	-	-	-	0.64 ± 0.05	-
C18:1 isomer n.d.	-	-	-	-	0.63 ± 0.02	-
C20:1 n-9 cis	1.19 ± 0.05	0.13 ± 0.02	0.09 ± 0.01	-	0.69 ± 0.00	0.29 ± 0.03
C22:1 n-9 cis	0.76 ± 0.02	-	-	-	0.39 ± 0.00	-
C24:1 n-9 cis	0.13 ± 0.01	-	-	-	-	-
Total MUFA	63.66	34.20	24.59	0.52	89.57	50.68
C16:2 n-6	-	0.22 ± 0.00	0.22 ± 0.00	-	-	0.20 ± 0.01
C18:2 n-4	-	0.17 ± 0.01	0.13 ± 0.01	-	-	-
C18:2 n-6 (9c,11c)	19.19 ± 0.06	1.08 ± 0.04	-	0.19 ± 0.00	0.81 ± 0.02	11.97 ± 0.20
C18:2 n-6 (9c, 11t) CLA	-	0.77 ± 0.03	1.60 ± 0.04	-	-	-
C18:2 isomer n.d.	-	-	-	-	0.46 ± 0.04	-
C18:2 isomer n.d.	-	-	-	-	0.67 ± 0.02	-
C18:2 isomer n.d.	-	-	-	-	0.52 ± 0.11	-
C18:2 isomer n.d.	-	-	-	-	0.19 ± 0.02	-
C18:2 isomer n.d.	-	-	-	-	0.22 ± 0.02	-
C18:2 isomer n.d.	-	-	-	-	0.08 ± 0.01	-
C18:3 n-3 *cis*	7.20 ± 0.09	0.75 ± 0.01	0.31 ± 0.02	-	0.62 ± 0.00	4.97 ± 0.12
C18:3 n-6 *cis*	-	0.13 ± 0.00	0.16 ± 0.00	-	-	-
C20:2 n-6 *cis*	-	-	-	-	0.10 ± 0.00	-
C20:3 n-6 *cis*	-	0.15 ± 0.02	0.09 ± 0.01	-	0.32 ± 0.00	-
C20:4 n-6 *cis*	-	0.13 ± 0.00	0.16 ± 0.00	-	-	-
Total PUFA	26.39	4.14	3.02	0.19	3.99	17.14
Total n-6	19.19	2.48	2.23	0.19	3.37	12.17
Total n-3	7.20	0.75	0.31	0.00	0.62	4.97
Ratio n-6/n-3	2.66	3.31	7.19	-	5.43	2.49
LA/ALA	2.66	2.46	5.16	-	1.31	2.40
PUFA/SFA	2.65	0.07	0.04	0.01	0.62	0.53
Total FA	100.01	99.99	100.00	100.01	100.01	99.99

Samples: rapeseed oil (RO), anhydrous milk fat (AMF and AMF41), mono- and diacylglycerols (PS and DMG), and blend (RO (58%), AMF (28%), AMF41 (12%), DMG (1.4%) and PS (0.6%)). SFA: saturated fatty acids; MUFA: monounsaturated fatty acids; PUFA: polyunsaturated fatty acids; CLA: conjugated linoleic acid; n.d.: not determined; LA: linoleic acid; ALA: linolenic acid. Results expressed as percentage of total FA.

**Table 2:** Triacylglycerols profiles.

ECN ^1^	TAG (%)	AMF	AMF41	Blend	ECN	TAG (%)	RO
28	BuCyO	2.01 ± 0.09	1.63 ± 0.31	1.12 ± 0.27			
30	BuCO	1.43 ± 0.01	1.03 ± 0.18	0.68 ± 0.12			
30	BuCP	1.25 ± 0.18	0.99 ± 0.18	0.66 ± 0.18			
32	CoCP	0.93 ± 0.15	0.74 ± 0.17	1.22 ± 0.11			
32	BuLaO	2.23 ± 0.18	1.32 ± 0.08	0 85 ± 0.06			
32	CyCyP CyCyL	2.00 ± 0.19	1.47 ± 0.04	1.01 ± 0.12			
32	BuLaP	2.24 ± 0.12	1.47 ± 0.37	1.51 ± 0.13			
34	BuMO	4.47 ± 0.20	2.96 ± 0.12	1.59 ± 0.06			
34	BuMP	4.40 ± 0.25	3.38 ± 0.30	1.81 ± 0.09	40	LnLL	0.61 ± 0.20
36	BuOO	5.10 ± 0.34	3.38 ± 0.17	2.42 ± 0.03	40	OLnLn	2.73 ± 0.02
36	BuPO	6.72 ± 0.40	4.65 ± 0.07	2.40 ± 0.24	42	LLL	0.32 ± 0.12
36	BuPP CoMP	6.63 ± 0.75	5.67 ± 0.23	0.80 ± 0.04			
38	CoPO CyMO	2.11 ± 0.76	2.34 ± 0.14	1.60 ± 0.35			
38	CoPP	2.27 ± 0.71	0.98 ± 0.33	0.84 ± 0.18			
38	BuPS	4.00 ± 0.30	3.09 ± 0.17	1.42 ± 0.32			
40	CMO CyPO	3.68 ± 0.28	2.53 ± 0.05	1.52 ± 0.21			
40	CMP	1.72 ± 0.25	1.23 ± 0.25	0.98 ± 0.05			
40	CoPS CyPP	2.94 ± 0.26	3.57 ± 0.45	5.08 ± 0.08	42	LOLn	7.32 ± 0.21
42	COO	2.25 ± 0.19	1.48 ± 0.14	1.68 ± 0.24	42	LPLn	0.91 ± 0.20
42	CPO	3.85 ± 0.14	3.24 ± 0.20	1.32 ± 0.24			
42	CPP CMS	2.81 ± 0.15	4.58 ± 0.24	11.98 ± 0.16	44 44	OLL LnOO	4.71 ± 0.56 14.12 ± 0.40
44	LaPO	2.53 ± 0.06	1.87 ± 0.05	2.28 ± 0.08	44	LLP	2.20 ± 0.40
44	MMP LaPP	3.36 ± 0.11	3.33 ± 0.26	1.64 ± 0.24			
46	OOL	2.82 ± 0.20	5.85 ± 0.11	13.86 ± 0.59	46	LOO	22.51 ± 0.93
46	MOO SOLn	3.83 ± 0.49	2.99 ± 0.23	4.66 ± 0.15	46	LOP	4.71 ± 0.55
46	MPO MPP	3.91 ± 0.66	6.32 ± 0.43	2.55 ± 0.05	46	SPLn	0.61 ± 0.21
48	OOO	2.78 ± 0.52	6.75 ± 0.11	17.72 ± 0.75	48	OOO	29.38 ± 0.61
48	POO	4.67 ± 0.55	3.48 ± 0.27	5.52 ± 0.15	48	OOP	5.84 ± 0.36
48	POP	4.53 ± 0.46	6.98 ± 1.08	2.92 ± 0.07	48	OPP	1.40 ± 0.30
50	SOO	1.22 ± 0.20	5.44 ± 0.73	2.51 ± 0.09			
50	MSS POS SSL	1.99 ± 0.20	1.49 ± 0.44	2.36 ± 0.20	50	POS	2.59 ± 0.11
50	PPS OSS	3.31 ± 0.07	3.77 ± 0.33	1.50 ± 0.20			

Samples: rapeseed oil (RO), anhydrous milk fat (AMF and AMF41), mono- and diacylglycerols (PS and DMG) and blend (RO (58%), AMF (28%), AMF41 (12%), DMG (1.4%) and PS (0.6%)). Bu: butyric acid (C4:0); Ca: caproic acid (C6:0); Cy: caprylic acid (C8:0); C: capric acid (C10:0); La: lauric acid (C12:0); M: myristic acid (C14:0); P: palmitic acid (C16:0); S: stearic acid (C18:0); O: oleic acid (C18:1 n-9); L: linoleic acid (C18:2 n-6); Ln: linolenic acid (C18:3 n-3). Results expressed as percentage of total TAG. ^1^ Equivalent carbon number.

**Table 3:** Peak temperatures and enthalpies of melting and crystallization (n=3).

	Exothermic Profile				Endothermic Profile			
	T_c_ (°C)	T_onset_ (°C)	T_offset_ (°C)	ΔH (J g^−1^)	T_m_ (°C)	T_onset_ (°C)	T_offset_ (°C)	ΔH (J g^−1^)
RO	−54.99 ± 0.41	−18.58 ± 0.32	−72.02 ± 1.73	51.68 ± 3.08	−18.87 ± 0.19	−35.05 ± 0.51	−5.93 ± 0.43	64.38 ± 1.84
AMF	4.62 ± 0.14	13.57 ± 0.96	−38.42 ± 1.93	55.72 ± 0.31	12.95 ± 0.33	−23.80 ± 0.16	33.59 ± 0.64	61.96 ± 2.51
AMF41	21.83 ± 0.67	26.90 ± 1.61	−28.39 ± 0.18	71.12 ± 1.21	38.55 ± 0.00	−12.98 ± 0.84	44.74 ± 0.30	76.14 ± 1.36
PS	53.73 ± 0.56	58.10 ± 0.73	25.42 ± 0.83	136.46 ± 1.82	59.76 ± 0.51	23.96 ± 0.46	66.32 ± 1.16	134.93 ± 2.63
DMG	29.91 ± 0.84	34.15 ± 1.01	−28.54 ± 0.83	70.88 ± 3.84	49.46 ± 0.37	16.43 ± 0.28	56.48 ± 0.46	69.08 ± 0.72
Blend	−57.88 ± 0.36	14.41 ± 1.42	−71.80 ± 0.72	65.26 ± 2.15	7.21 ± 0.81	−38.28 ± 0.10	36.08 ± 0.09	76.38 ± 2.97

Samples: rapeseed oil (RO), anhydrous milk fat (AMF and AMF41), mono- and diacylglycerols (PS and DMG) and blend (RO (58%), AMF (28%), AMF41 (12%), DMG (1.4%), and PS (0.6%)). T_c_: crystallization temperature of peak; T_m_: melting temperature of peak; T_onset_: onset peak temperature; T_offset_: offset peak temperature; ΔH: enthalpy. Blend: RO (58.28%), AMF (27.68%), AMF41 (12.08%), DMG (1.36%), and PS (0.58%).

**Table 4:** Solid fat content measurements by p-NMR and DSC at different temperatures (n = 3).

	p-NMR (%)			
T °C	10 °C	20 °C	30 °C	35 °C
AMF	42.40 ± 0.24 ^a,A^	13.89 ± 0.16 ^b,A^	2.71 ± 0.18 ^c,A^	0.23 ± 0.16 ^d,A^
AMF41	67.99 ± 0.01 ^a,B^	44.36 ± 0.08 ^b,B^	23.29 ± 0.08 ^c,B^	13.40 ± 0.12 ^d,B^
Blend	15.50 ± 0.28 ^a,C^	6.54 ± 0.07 ^b,C^	1.53 ± 0.31 ^c,C^	0.20 ± 0.16 ^d,C^
	**DSC (%)**			
T °C	10 °C	20 °C	30 °C	35 °C
AMF	49.07 ± 1.26 ^a,A^	14.87 ± 0.16 ^b,A^	1.10 ± 0.10 ^c,A^	0.01 ± 0.01 ^d^
AMF41	78.06 ± 1.00 ^a,B^	69.37 ± 0.16 ^b,B^	44.32 ± 0.38 ^c,B^	26.16 ± 0.33 ^d,B^
Blend	25.02 ± 0.49 ^a,C^	12.16 ± 0.15 ^C^	1.59 ± 0.20 ^C^	0.05 ± 0.05

Samples: anhydrous milk fat (AMF and AMF41) and blend (RO (58%), AMF (28%), AMF41 (12%), DMG (1.4%), and PS (0.6%)). p-NMR: pulsed nuclear magnetic resonance; DSC: differential scanning calorimetry; T °C: temperature. Results expressed in % of total fat content. a–d: Significance (*p* < 0.05) between samples to AMF, at a given temperature. A–C: Significance (*p* < 0.05) within each sample for consecutive temperature.

**Table 5:** Raman peak positions and the respective vibrational modes [adapted from [65,71]].

Wave Number cm^−1^	Functional Groups	Mode of Vibration	Assignment	Component
1746	C=O (R-C=OOR)	Carbonyl stretching	υ(C=O)	Fatty acids
1659	C=C (R-HC=CH-R)	Olefinic stretching	υ(C=C)	Unsaturated fatty acids
1442	CH (-CH_2_)	Methylene scissoring	δ(CH_2_)_sc_	Saturated fatty acids
1298	CH (-CH_2_)	Methylene twisting	δ(CH_2_)_tw_	Saturated fatty acids
1267	=CH (R-CH=CH-R)	In-plane olefinic hydrogen deformation	δ(=CH)_ip_	Unsaturated fatty acids
1131	C-C (CH_2_)n	In-phase aliphatic stretching all-*trans*	υ(C-C)_ip_	Extended chains
1083	C-C (CH_2_)n	In-phase aliphatic stretching in *gauche*	υ(C-C)_g_	Extended chains
1065	C-C (CH_2_)n	Out-of-phase aliphatic stretching all-*trans*	υ(C-C)_op_	Extended chains
971	C=C (R-HC=CH-R)	*trans* deformation	δ(C=C)	Unsaturated fatty acids
869	C-C (CH_2_)n	Stretching	υ(C-C)	Extended chains

υ: stretching, δ: deformation, sc: scissoring, tw: twisting, ip: in-plane or in-phase, g: gauche, op: out-of-phase.

**Table 6:** Avrami parameters obtained from fitting experimental data exotherm during 180-min isotherm.

Samples	n ^1^	k ^2^ (min^−n^)	t_1/2_ ^3^ (min)
AMF	2.30	1.99 × 10^−3^	22.58
AMF41	2.38	8.53 × 10^−4^	8.00
DMG	4.00	1.07 × 10^−7^	37.58
Blend	2.22	7.99 × 10^−3^	10.63

Samples: anhydrous milk fat (AMF and AMF41), mono- and diacylglycerols (PS and DMG) and blend (RO (58%), AMF (28%), AMF41 (12%), DMG (1.4%) and PS (0.6%)). ^1^ Avrami exponent; ^2^ Rate constant; ^3^ Half-time of crystallization.

## Data Availability

The original contributions presented in the study are included in the article, further inquiries can be directed to the corresponding author.
